# An active-set algorithm for solving large-scale nonsmooth optimization models with box constraints

**DOI:** 10.1371/journal.pone.0189290

**Published:** 2018-01-02

**Authors:** Yong Li, Gonglin Yuan, Zhou Sheng

**Affiliations:** 1 Guangxi Colleges and Universities Key Laboratory of Mathematics and Its Applications, College of Mathematics and Information Science, Guangxi University, Nanning, Guangxi 530004, China; 2 Department of Mathematics, Baise University, Baise, Guangxi, 533000, China; Universita degli Studi di Catania, ITALY

## Abstract

It is well known that the active set algorithm is very effective for smooth box constrained optimization. Many achievements have been obtained in this field. We extend the active set method to nonsmooth box constrained optimization problems, using the Moreau-Yosida regularization technique to make the objective function smooth. A limited memory BFGS method is introduced to decrease the workload of the computer. The presented algorithm has these properties: (1) all iterates are feasible and the sequence of objective functions is decreasing; (2) rapid changes in the active set are allowed; (3) the subproblem is a lower dimensional system of linear equations. The global convergence of the new method is established under suitable conditions and numerical results show that the method is effective for large-scale nonsmooth problems (5,000 variables).

## Introduction

Consider

$$\begin{array}{c} \min_{x\in K}f\left(x\right), \end{array}$$ (1)

where *f*: ℜ^*n*^ → ℜ is a possibly nonsmooth convex function, *K* = {*x* ∣ *l* ≤ *x* ≤ *u*}, the vectors *l* and *u* represent lower and upper bounds on the variables, and *n* is the number of variables. Similar problems are discussed by Fukushima [1, 2], in which equality constraints are considered and a penalty strategy is used. The form of problem (1) can be viewed as an extension of the linearly constrained convex nonsmooth problem considered in, e.g., [3, 4] from linear to possibly nonlinear. In fact, many fields including finance, engineering, management, biology, and medicine can convert to the optimization models (1) (see [5–9] in detail).

Generally, nonsmooth problems are very difficult to solve even when they are unconstrained. Derivative-free methods, like Powell’s method [10] or genetic algorithms [11], may be unreliable and become inefficient whenever the dimension of the problem increases. The direct application of smooth gradient-based methods to nonsmooth problems may lead to a failure in optimality conditions, in convergence, or in gradient approximation [12]. Wolfe [13] and Lemaréchal [14] initiated a giant stride forward in nonsmooth optimization by the bundle concept. Kiwiel [15] proposed a bundle variant that is close to the bundle trust iteration method [16]. Some good results about the bundle technique can be found in [17–19] etc. At the moment, various versions of bundle methods are regarded as the most effective and reliable methods for nonsmooth optimization. Bundle methods are efficient for small- and medium-scale problems. This is explained by the fact that bundle methods need relatively large bundles to be capable of solving the problems efficiently [17]. Therefore, special tools for solving nonsmooth optimization problems are needed. At present, Haarala et al. (see [20, 21] etc.) introduce the limited memory bundle methods for large scale nonsmooth unconstrained and constrained minimization, which are a hybrid of the variable metric bundle methods and the limited memory variable metric methods and some good results are obtained. More related literature can be found in [22–26]. The test problems can have thousands of decision variables. Yuan et al. [27–31] make some studies where nonsmooth problems with the largest dimension 100,000 were solved in the unconstrained cases [28].

The active-set method can be generalized easily when the objective function is nonsmooth. For example, Sreedharan [32] extends the method developed in [33] to solve nonsmooth problems with a special objective function and inequality constraint. Also, it is quite easy to generalize the *ε*-active set method to the nondifferentiable case (see, e.g., [34]). In this paper we use the active-set method to solve (1) when the objective function *f* is convex but not necessarily differentiable. Convexity, which is not essential for our study, is assumed only for simplicity. For the objective function, we first use the Moreau-Yosida regularization technique to make it smooth. Then the active-set limited memory BFGS (L-BFGS) technique is proposed to solve it. Global convergence is established under suitable conditions. The main features of the proposed method are as follows.

1. The iterates are feasible; large changes are allowed in the active set; the subproblem has lower dimension; and the objective function sequence {*f*^*MY*^ (*x*_*k*_, *ε*_*k*_)} is decreasing.
2. The L-BFGS method uses function and gradient values.
3. Global convergence is established under suitable conditions.
4. Numerical results show that the method is effective for large-scale problems (up to 5,000 variables).

The paper is organized as follows. In the next section, we briefly review some nonsmooth analysis, a BFGS method and the L-BFGS method for unconstrained optimization, and the motivation for using these techniques. In Section 3, we describe the active-set algorithm with L-BFGS update for (1). In Section 4, global convergence is established under suitable conditions. Numerical results are reported in Section 5, and conclusions are given in the last section.

## Nonsmooth analysis and the L-BFGS update

This section states some results on nonsmooth analysis, a modified BFGS formula, and a L-BFGS formula for unconstrained optimization problems.

### Some results of convex analysis and nonsmooth analysis

Let *f*^*MY*^: ℜ^*n*^ → ℜ be the so-called Moreau-Yosida regularization of *f* defined by

$$\begin{array}{c} f^{MY}\left(x\right)=\min_{z\in {ℜ}^{n}}\{f\left(z\right)+\frac{1}{2\lambda }{∥z-x∥}^{2}\}, \end{array}$$ (2)

where ‖⋅‖ denotes the Euclidean norm of vectors and λ is a positive parameter. Then it is not difficult to see that problem (1) is equivalent to the problem

$$\begin{array}{c} \min_{x\in K}f^{MY}\left(x\right). \end{array}$$ (3)

The function *f*^*MY*^ is a differentiable convex function and has a Lipschitz continuous gradient even when *f* is nondifferentiable. Under some reasonable conditions, using the following properties of *f*^*MY*^ (*x*) and assuming ∇*f*^*MY*^ (*x*) is globally Lipschitz continuous, the gradient ∇*f*^*MY*^ (*x*) is semismooth (see [35, 36] etc.). By these properties, many algorithms have been given for solving (3) (see [37] etc.) when *K* = ℜ^*n*^. Some features of *f*^*MY*^ (*x*) can be seen in [38–40] et al. Set

$$\begin{array}{c} \theta \left(z\right)=f\left(z\right)+\frac{1}{2\lambda }{∥z-x∥}^{2} \end{array}$$

and denote *p*(*x*) = argmin *θ*(*z*). Since *θ*(*z*) is strongly convex, it is easy to deduce that *p*(*x*) is well-defined and unique. Then *f*^*MY*^ (*x*) in (2) can be rewritten as

$$\begin{array}{c} f^{MY}\left(x\right)=f\left(p\left(x\right)\right)+\frac{1}{2\lambda }{∥p\left(x\right)-x∥}^{2}. \end{array}$$

The generalized Jacobian of *f*^*MY*^ (*x*) and the property of BD-regular can be found in [41, 42], respectively. Here some properties are listed without proof.

(i) The function *f*^*MY*^ is finite-valued, convex, and everywhere differentiable. If *g*(*x*) = ∇*f*^*MY*^ (*x*), then *g*: ℜ^*n*^ → ℜ^*n*^ is globally Lipschitz continuous:

$$\begin{array}{c} ∥g\left(x\right)-g\left(y\right)∥\leq \frac{1}{\lambda }∥x-y∥,\;\;\forall \;\;x,y\in {ℜ}^{n}, \end{array}$$ (4)

where

$$\begin{array}{c} g\left(x\right)=\nabla f^{MY}\left(x\right)=\frac{x-p(x)}{\lambda }. \end{array}$$ (5)

(ii) *g* is BD-regular at *x* means that all matrices *V* ∈ ∂_*B*_*g*(*x*) are nonsingular. Then there exist constants *μ*_1_ > 0, *μ*_2_ > 0 and a neighborhood Ω of *x* satisfying

$$\begin{array}{c} d^{T}Vd\geq \mu_{1}{∥d∥}^{2},\;∥V^{-1}∥\leq \mu_{2},\;\forall \;\;d\in {ℜ}^{n},\;V\in \partial_{B}g\left(x\right). \end{array}$$

It is easy to find that *p*(*x*) of the minimizer for *θ*(*z*) is difficult or even impossible to solve exactly. Fortunately, for each *x* ∈ ℜ^*n*^ and any *ε* > 0, there exists a vector *p*(*x*, *ε*) ∈ ℜ^*n*^ satisfying

$$\begin{array}{c} f^{MY}\left(p\left(x,\varepsilon \right)\right)+\frac{1}{2\lambda }{∥p\left(x,\varepsilon \right)-x∥}^{2}\leq f^{MY}\left(x\right)+\varepsilon . \end{array}$$ (6)

Thus, we can use *p*(*x*, *ε*) to define approximations of *f*^*MY*^ (*x*) and *g*(*x*) by

$$\begin{array}{c} f^{MY}\left(x,\varepsilon \right)=f\left(p\left(x,\varepsilon \right)\right)+\frac{1}{2\lambda }{∥p\left(x,\varepsilon \right)-x∥}^{2}, \end{array}$$ (7)

and

$$\begin{array}{c} g\left(x,\varepsilon \right)=\frac{x-p(x,\varepsilon )}{\lambda }, \end{array}$$ (8)

respectively. Some implementable algorithms to find such *p*(*x*, *ε*) for a nondifferentiable convex function are introduced in [43]. A remarkable feature of *f*^*MY*^ (*x*, *ε*) and *g*(*x*, *ε*) given by [35] is introduced, which show that, by choosing parameter *ε* small enough, we can compute approximations *f*^*MY*^ (*x*, *ε*) and *g*(*x*, *ε*) closing to *f*^*MY*^ (*x*) and *g*(*x*) respectively.

**Proposition 1.** *Suppose that* *f*^*MY*^ (*x*, *ε*) *and* *g*(*x*, *ε*) *are defined by* (7) and (8), *respectively*. *Let* *p*(*x*, *ε*) *be a vector satisfying* (6). *Then*

$$\begin{array}{c} f^{MY}\left(x\right)\leq f^{MY}\left(x,\varepsilon \right)\leq f^{MY}\left(x\right)+\varepsilon , \end{array}$$ (9)

$$\begin{array}{c} ∥p\left(x,\varepsilon \right)-p\left(x\right)∥\leq \sqrt{2\lambda \varepsilon }, \end{array}$$ (10)

*and*

$$\begin{array}{c} ∥g\left(x,\varepsilon \right)-g\left(x\right)∥\leq \sqrt{2\varepsilon /\lambda } \end{array}$$ (11)

*hold*.

### A modified BFGS formula and the L-BFGS formula

The BFGS method is one of the most effective quasi-Newton methods for unconstrained optimization problems (UNP) min_*x*∈ℜ^*n*^_ *h*(*x*), where *h*(*x*): ℜ^*n*^ → ℜ is continuously differentiable. The famous BFGS quasi-Newton formula is

$$\begin{array}{c} B_{k+1}=B_{k}-\frac{B_{k}s_{k}s_{k}^{T}B_{k}}{s_{k}^{T}B_{k}s_{k}}+\frac{y_{k}y_{k}^{T}}{s_{k}^{T}y_{k}}, \end{array}$$ (12)

where *s*_*k*_ = *x*_*k*+1_ − *x*_*k*_, *y*_*k*_ = ∇*h*(*x*_*k*+1_) − ∇*h*(*x*_*k*_), and it is easy to see that the quasi-Newton equation

$$\begin{array}{c} B_{k+1}s_{k}=y_{k} \end{array}$$ (13)

holds. If *H*_*k*_ is the inverse of *B*_*k*_, we get the inverse update formula of (12):

$$\begin{array}{ccc} H_{k+1} & = & H_{k}-\frac{y_{k}^{T}\left(s_{k}-H_{k}y_{k}\right)s_{k}s_{k}^{T}}{{\left(y_{k}^{T}s_{k}\right)}^{2}}+\frac{\left(s_{k}-H_{k}y_{k}\right)s_{k}^{T}+s_{k}{\left(s_{k}-H_{k}y_{k}\right)}^{T}}{{\left(y_{k}^{T}s_{k}\right)}^{2}}\\ & = & \left(I-\frac{s_{k}y_{k}^{T}}{y_{k}^{T}s_{k}}\right)H_{k}\left(I-\frac{y_{k}s_{k}^{T}}{y_{k}^{T}s_{k}}\right)+\frac{s_{k}s_{k}^{T}}{y_{k}^{T}s_{k}}, \end{array}$$ (14)

which is the dual form of the DFP update formula in the sense that *H*^*k*^ ↔ *B*^*k*^, *H*^*k*+1^ ↔ *B*^*k*+1^, and *s*^*k*^ ↔ *y*^*k*^. The L-BFGS method is an adaptation of the BFGS method to large-scale problems (see [44–46] in detail). Instead of storing the matrices *H*^*k*^, at every iteration *x*_*k*_ the method stores a small number, say *m*, of correction pairs {*s*_*i*_, *y*_*i*_}, *i* = *k* − 1, …, *k* − *m*. Let $\rho_{k}=\frac{1}{y_{k}^{T}s_{k}}$ and $V_{k}=I-\rho_{k}y_{k}s_{k}^{T}$. The L-BFGS update has the form

$$\begin{array}{ccc} H_{k+1} & = & V_{k}^{T}\left[V_{k-1}^{T}H_{k-1}V_{k-1}+\rho_{k-1}s_{k-1}s_{k-1}^{T}\right]V_{k}+\rho_{k}s_{k}s_{k}^{T}\\ & = & V_{k}^{T}V_{k-1}^{T}H_{k-1}V_{k-1}+V_{k}^{T}\rho_{k-1}s_{k-1}s_{k-1}^{T}V_{k}+\rho_{k}s_{k}s_{k}^{T}\\ & = & \cdot \cdot \cdot \\ & = & \left[V_{k}^{T}\cdots V_{k-m+1}^{T}\right]H_{k-m+1}\left[V_{k-m+1}\cdots V_{k}\right]+\rho_{k+m-1}[V_{k-1}^{T}\\ & \cdots & V_{k-m+2}^{T}]s_{k-m+1}s_{k-m+1}^{T}\left[V_{k-m+2}...V_{k-1}\right]+\cdots +\rho_{k}s_{k}s_{k}^{T}, \end{array}$$ (15)

which can provide a fast rate of linear convergence and requires minimal storage. From the BFGS formula (14) and the L-BFGS update (15), it is not difficult to find that both of these formulas contain only the gradient information of the objective function, while the function values available are neglected. Some modified quasi-Newton formulas using both gradient and function information are presented (e.g. [47, 48]). Wei et al. [49] also gave a new quasi-Newton equation

$$\begin{array}{c} B_{k+1}s_{k}=y_{k}+A_{k}s_{k}, \end{array}$$

where $A_{k}=\frac{2\left[h\left(x_{k}\right)-h\left(x_{k}+\alpha_{k}d_{k}\right)\right]+{\left(\nabla h\left(x_{k}+\alpha_{k}d_{k}\right)+\nabla h\left(x_{k}\right)\right)}^{T}s_{k}}{∥s_{k}{∥}^{2}}$ and the corresponding BFGS update formula is defined by

$$\begin{array}{c} B_{k+1}=B_{k}-\frac{B_{k}s_{k}s_{k}^{T}B_{k}}{s_{k}^{T}B_{k}s_{k}}+\frac{y_{k}^{*}{\left(y_{k}^{*}\right)}^{T}}{s_{k}^{T}y_{k}^{*}}, \end{array}$$ (16)

where $y_{k}^{*}=y_{k}+A_{k}s_{k}$. The quasi-Newton formula (16) contains both gradient and function information; moreover the modified BFGS update formula possesses a higher order approximation of ∇^2^*h*(*x*) than that of the standard BFGS update (see [47, 49] in detail).

Global convergence and superlinear convergence of the quasi-Newton method with (16) have been established for uniformly convex functions [47, 49], but fails for general convex functions. One of the main reasons lies in the condition $s_{k}^{T}y_{k}>0$ that may not hold for general convex functions. To overcome the weaknesses, Yuan and Wei [50] presented a modified $y_{k}^{*}$ named $y_{k}^{1*}=y_{k}+\max\left\{0,A_{k}\right\}s_{k}$. The idea of paper [50] is based on the following two cases:

**Case i:** It follows from *A*^*k*^ > 0 that
  $$\begin{array}{c} s_{k}^{T}\left(y_{k}+\frac{A_{k}}{∥s_{k}{∥}^{2}}s_{k}\right)={\left(s_{k}\right)}^{T}y_{k}+A_{k}>s_{k}^{T}y_{k}\geq 0. \end{array}$$ (17)
**Case ii:** On the other hand, if *A*^*k*^ < 0, it is easy to get
  $$\begin{array}{ccc} 0 & > & A_{k}=\frac{2\left[h\left(x_{k}\right)-h\left(x_{k}+\alpha_{k}d_{k}\right)\right]+{\left(\nabla h\left(x_{k}+\alpha_{k}d_{k}\right)+\nabla h\left(x_{k}\right)\right)}^{T}s_{k}}{∥s_{k}{∥}^{2}}\\ & \geq & \frac{-2\nabla h{\left(x_{k}+\alpha_{k}d_{k}\right)}^{T}s_{k}+{\left(\nabla h\left(x_{k}+\alpha_{k}d_{k}\right)+\nabla h\left(x_{k}\right)\right)}^{T}s_{k}}{∥s_{k}{∥}^{2}}\\ & = & \frac{-s_{k}^{T}y_{k}}{∥s_{k}{∥}^{2}}, \end{array}$$ (18)
  where the second inequality follows the definition of the convexity of *h*(*x*), which means that $s_{k}^{T}y_{k}>0$ holds. This modified BFGS formula with $y_{k}^{1*}$ possesses global convergence and superlinear convergence for general convex functions. However, its applications in L-BFGS and nonsmooth optimization have not been widely studied.

This article will attempt to do this. The following gives the modified L-BFGS formula for (3) with form

$$\begin{array}{ccc} H_{k+1} & = & {\left(V_{k}^{*}\right)}^{T}\left[{\left(V_{k-1}^{*}\right)}^{T}H_{k-1}V_{k-1}^{*}+\rho_{k-1}^{*}s_{k-1}s_{k-1}^{T}\right]V_{k}^{*}+\rho_{k}^{*}s_{k}s_{k}^{T}\\ & = & {\left(V_{k}^{*}\right)}^{T}{\left(V_{k-1}^{*}\right)}^{T}H_{k-1}V_{k-1}^{*}+{\left(V_{k}^{*}\right)}^{T}\rho_{k-1}^{*}s_{k-1}s_{k-1}^{T}V_{k}^{*}+\rho_{k}^{*}s_{k}s_{k}^{T}\\ & = & \cdot \cdot \cdot \\ & = & \left[{\left(V_{k}^{*}\right)}^{T}\cdots {\left(V_{k-m+1}^{*}\right)}^{T}\right]H_{k-m+1}\left[V_{k-m+1}^{*}\cdots V_{k}^{*}\right]+\rho_{k+m-1}^{*}[{\left(V_{k-1}^{*}\right)}^{T}\\ & \cdots & {\left(V_{k-m+2}^{*}\right)}^{T}]s_{k-m+1}{\left(s_{k-m+1}\right)}^{T}\left[V_{k-m+2}^{*}...V_{k-1}^{*}\right]+\cdots +\rho_{k}^{*}s_{k}s_{k}^{T}, \end{array}$$ (19)

where $\rho_{k}^{*}=\frac{1}{{\left(\delta_{k}^{*}\right)}^{T}s_{k}},\;\;V_{k}^{*}=I-\rho_{k}^{*}\delta_{k}^{*}s_{k}^{T}$, $\delta_{k}^{*}=\delta_{k}+\max\left\{A_{k}^{2*},0\right\}s_{k}$, *δ*_*k*_ = *g*(*x*_*k*+1_, *ε*_*k*+1_) − *g*(*x*_*k*_, *ε*_*k*_), and $A_{k}^{2*}=\frac{2\left(f^{MY}\left(x_{k},\varepsilon_{k}\right)-f^{MY}\left(x_{k}+\alpha_{k}d_{k},\varepsilon_{k+1}\right)\right)+{\left(g\left(x_{k}+\alpha_{k}d_{k},\varepsilon_{k+1}\right)+g\left(x_{k},\varepsilon_{k}\right)\right)}^{T}s_{k}}{∥s_{k}{∥}^{2}}$. It is clear that the modified L-BFGS formula (19) contains both function and gradient information at the current and previous step if $A_{k}^{2*}>0$. In the following, the matrix *H*_*k*_ is generated by (19). This is very costly for even moderately large nonsmooth problems with box constraints, since the limited memory update is used to store ${\left\{s_{k},\delta_{k}^{*}\right\}}_{k-m+1}^{k}$, update it as a full matrix, reduce in the free subspace, and the set of active constraints changes at the first finite steps.

Inspired by the Moreau-Yosida regularization and the modified method of [50], we combine them with the limited memory technique, and use them to solve box constrained optimization with nonsmooth objective function. This paper can be regarded as an improvement of the method in [51] with extension to nonsmooth objective functions. Comparing with [51], at each step of our method, a lower-dimensional system of nonlinear equations and nonsmooth objective function needs to be solved. The method is also similar to the algorithm in [44], but, at each iteration, we use an identification technique and solve nonsmooth optimization problems.

## L-BFGS active-set algorithm

The following assumptions are needed to obtain convergence.

**Assumption A** The level set *ϕ* = {*x* ∈ ℜ^*n*^ ∣ *f*^*MY*^ (*x*) ≤ *f*^*MY*^ (*x*_0_)} ⋂ *K* is compact.

**Assumption B** *f*^*MY*^ is bounded from below and the sequence {*ε*_*k*_} converges to zero. We first solve (3) and adapt its solution to problem (1). With the feasible region *K* = {*x* ∈ ℜ^*n*^: *l*_*i*_ ≤ *x*_*i*_ ≤ *u*_*i*_, *i* = 1, …, *n*}, a vector $\bar{x}\in K$ is said to be a stationary point for problem (3) if the relations

$$\{\begin{array}{lll} l_{i}={\bar{x}}_{i} & \Rightarrow & g_{i}\left(\bar{x}\right)\geq 0,\\ l_{i}<{\bar{x}}_{i}<u_{i} & \Rightarrow & g_{i}\left(\bar{x}\right)=0,\\ {\bar{x}}_{i}=u_{i} & \Rightarrow & g_{i}\left(\bar{x}\right)\leq 0, \end{array}$$ (20)

hold. Consider the relations

$$\{\begin{array}{lll} l_{i}={\bar{x}}_{i} & \Rightarrow & g_{i}\left(\bar{x},\bar{\varepsilon }\right)\geq 0,\\ l_{i}<{\bar{x}}_{i}<u_{i} & \Rightarrow & g_{i}\left(\bar{x},\bar{\varepsilon }\right)=0,\\ {\bar{x}}_{i}=u_{i} & \Rightarrow & g_{i}\left(\bar{x},\bar{\varepsilon }\right)\leq 0, \end{array}$$ (21)

where the scalar $\bar{\varepsilon }\in ℜ$ tends to zero. By the definition of $g(\bar{x})$ and $g(\bar{x},\bar{\varepsilon })$, we have

$$\begin{array}{ccc} g(\bar{x},\bar{\varepsilon }) & = & \frac{\bar{x}-p\left(\bar{x},\bar{\varepsilon }\right)}{\lambda }\\ & = & \frac{\bar{x}-p\left(\bar{x}\right)}{\lambda }+\frac{p\left(\bar{x}\right)-p\left(\bar{x},\bar{\varepsilon }\right)}{\lambda }\\ & = & g\left(\bar{x}\right)+\frac{p\left(\bar{x}\right)-p\left(\bar{x},\bar{\varepsilon }\right)}{\lambda }. \end{array}$$

By (10), if $\bar{\varepsilon }\to 0,$ we get

$$\begin{array}{c} g\left(\bar{x},\bar{\varepsilon }\right)=g\left(\bar{x}\right)+o\left(1\right). \end{array}$$

Thus, if (21) holds, it is easy to deduce that (20) holds. In the following, without special note, we concentrate on the relation (21) and regard it as the stationary point condition. In the following, we always suppose that the point *x*_*k*_ is consistent with *ε*_*k*_ and $\bar{x}$ is consistent with $\bar{\varepsilon }$ without special remark.

Similar to normal numerical optimization methods, the iteration formula is

$$\begin{array}{c} x_{k+1}=x_{k}+\alpha_{k}d_{k},\;\;k=0,1,2,\ldots , \end{array}$$ (22)

where {*x*_*k*_} ⊆ *K* = {*x* ∈ ℜ^*n*^: *l*_*i*_ ≤ *x*^*i*^ ≤ *u*_*i*_, *i* = 1, …, *n*}, *x*^*i*^ is the *i*th element of *x*, *d*_*k*_ is a descent direction of *f*^*MY*^ at *x*_*k*_, and *α*_*k*_ is a step length determined by the Armijo line search technique

$$\begin{array}{c} f^{MY}\left(x_{k}+\alpha_{k}d_{k},\varepsilon_{k+1}\right)\leq f^{MY}\left(x_{k},\varepsilon_{k}\right)+\sigma \alpha_{k}g{\left(x_{k},\varepsilon_{k}\right)}^{T}d_{k}, \end{array}$$ (23)

where $0<\sigma <\frac{1}{2}$, *α*^*k*^ = 2^−*i*^ with the smallest integer *i* = 0, 1, 2, …, and the sequence {*ε*_*k*_} satisfies *ε*_*k*_ > *ε*_*k*+1_ > 0. Before we give the direction definition, we introduce the procedure that estimates the active bounds. Suppose that $\bar{x}\in {ℜ}^{n}$ is a stationary point of problem (1). Let the associated active constraint set be

$$\begin{array}{c} \bar{\Upsilon }=\left\{i:l_{i}={\bar{x}}^{i}\right\},\;\;\bar{\Lambda }=\left\{i:{\bar{x}}^{i}=u_{i}\right\} \end{array}$$ (24)

and the set of free variables be

$$\begin{array}{c} \bar{\Gamma }=\left\{1,\ldots ,n\right\}\backslash \left(\bar{\Upsilon }\cup \bar{\Lambda }\right). \end{array}$$

Then condition (21) can be stated in the form

$$\{\begin{array}{ll} g^{i}\left(\bar{x},\bar{\varepsilon }\right)\geq 0 & \forall i\in \bar{\Upsilon },\\ g^{i}\left(\bar{x},\bar{\varepsilon }\right)=0 & \forall i\in \bar{\Gamma },\\ g^{i}\left(\bar{x},\bar{\varepsilon }\right)\leq 0 & \forall i\in \bar{\Lambda }, \end{array}$$ (25)

where $g^{i}\left(\bar{x},\bar{\varepsilon }\right)$ is the *i*th element of $g(\bar{x},\bar{\varepsilon })$. Let *a*_*i*_ and *b*_*i*_ be nonnegative continuous bounded from above on *K*, satisfying, if *x*^*i*^ = *l*_*i*_ or *x*^*i*^ = *u*_*i*_ then *a*_*i*_(*x*) > 0 or *b*_*i*_(*x*) > 0, respectively. Define the following approximation Υ(*x*), Γ(*x*) and Λ(*x*) to $\bar{\Upsilon },\bar{\Gamma }$ and $\bar{\Lambda }$, respectively:

$$\begin{array}{ccc} \Upsilon (x) & = & \{i:x^{i}\leq l_{i}+a_{i}\left(x\right)g^{i}\left(x,\varepsilon \right)\},\\ \Lambda (x) & = & \{i:x^{i}\geq u_{i}+b_{i}\left(x\right)g^{i}\left(x,\varepsilon \right)\},\\ \Gamma (x) & = & \{1,\ldots ,n\}\backslash (\Upsilon \cup \Lambda ). \end{array}$$ (26)

**Theorem 1.** *For any feasible* *x*, Υ(*x*) ∩ Λ(*x*) = ∅. *Furthermore*, *if* $\bar{x}$ *is a stationary point of problem* (3) *where strict complementarity holds*, *then there exists a neighborhood* Ψ *of* $\bar{x}$ *such that for every feasible point* *x* *in this neighborhood we have*

$$\begin{array}{c} \Upsilon \left(x\right)=\bar{\Upsilon },\;\;\;\;\Gamma \left(x\right)=\bar{\Gamma },\;\;\;\;\Lambda \left(x\right)=\bar{\Lambda }. \end{array}$$ (27)

**Proof.** For any feasible *x*, if *k* ∈ Υ(*x*), it is obvious that *g*_*k*_(*x*, *ε*) ≥ 0 holds. Suppose that *k* ∈ Λ(*x*); then we have *u*_*k*_ ≥ *x*_*k*_ ≥ *u*_*k*_ + *b*_*k*_(*x*)*g*_*k*_(*x*, *ε*) ≥ *u*_*k*_. This implies that *l*_*k*_ = *x*_*k*_ = *u*_*k*_ and *g*_*k*_(*x*, *ε*) = 0, which is a contradiction. Thus Υ(*x*) ∩ Λ(*x*) = ∅.

Now we prove the second conclusion. If $i\in \bar{\Upsilon }$, then by the definition of $\bar{\Upsilon }$, $g_{i}\left(\bar{x},\bar{\varepsilon }\right)\geq 0$. Since *a*_*i*_ is nonnegative, then $\bar{x_{i}}\leq l_{i}+a_{i}\left(\bar{x}\right)g_{i}\left(\bar{x},\bar{\varepsilon }\right)$. Since both *a*_*i*_ and *g*_*i*_ are continuous in $\bar{x}$, we deduce that *i* ∈ Υ(*x*). Thus we have $\bar{\Upsilon }\subseteq \Upsilon \left(x\right)$.

Otherwise if *i* ∈ Υ(*x*), then by the definition of Υ(*x*), *a*_*i*_(*x*)*g*_*i*_(*x*, *ε*) ≥ *x*_*i*_ − *l*_*i*_ ≥ 0. Since *a*_*i*_ is nonnegative, *g*_*i*_(*x*, *ε*) ≥ 0. Since *g*_*i*_ is continuous in $\bar{x}$, we deduce that $i\in \bar{\Upsilon }$. Thus we get $\Upsilon \left(x\right)\subseteq \bar{\Upsilon }$.

Therefore, we obtain $\Upsilon \left(x\right)=\bar{\Upsilon }$. Analogously, we can conclude that $\Gamma \left(x\right)=\bar{\Gamma }$ and $\Lambda \left(x\right)=\bar{\Lambda }$. □

This theorem proved that Υ(*x*), Γ(*x*) and Λ(*x*) are “good” estimates of $\bar{\Upsilon },\bar{\Gamma }$ and $\bar{\Lambda }$. The proof can also be found in [52].

The search direction $d_{k}=\left(d_{k}^{\Upsilon_{k}},d_{k}^{\Gamma_{k}},d_{k}^{\Lambda_{k}}\right)$ is chosen as

$$\begin{array}{c} d_{k}^{i}=\{\begin{array}{cc} l_{i}-x_{k}^{i},\;\;\;\; & \forall i\in \Upsilon_{k},\\ u_{i}-x_{k}^{i},\;\;\;\;\;\; & \forall i\in \Lambda_{k},\\ d_{k}^{\Gamma_{k}},\;\;\;\;\;\; & \forall i\in \Gamma_{k}, \end{array} \end{array}$$ (28)

where

$$\begin{array}{c} d_{k}^{\Gamma_{k}}=\alpha_{k}^{*}d_{u}^{\Gamma_{k}} \end{array}$$ (29)

with

$$\begin{array}{c} d_{u}^{\Gamma_{k}}=-{\bar{H}}_{k}g_{k}^{\Gamma_{k}} \end{array}$$ (30)

and

$$\begin{array}{c} \alpha_{k}^{*}=\max\left\{\alpha |\alpha \leq 1,l_{i}-x_{k}^{i}\leq \alpha d_{u}^{i}\leq u_{i}-x_{k}^{i},\;\;i\in \Gamma_{k}\right\}, \end{array}$$ (31)

Υ_*k*_ = Υ(*x*_*k*_), Γ_*k*_ = Γ(*x*_*k*_), Λ_*k*_ = Λ(*x*_*k*_), ${\bar{H}}_{k}=Z^{T}H_{k}Z\in {ℜ}^{|\Gamma_{k}\left|\times \right|\Gamma_{k}|}$ is an approximation to the reduced inverse Hessian matrix, *H*_*k*_ is an approximation of the full space inverse Hessian matrix, *Z* is the matrix whose columns are {*e*_*i*_ ∣ *i* ∈ Γ_*k*_}, and *e*_*i*_ is the *i*th column of the identity matrix in ℜ^*n*×*n*^. If the strict complementarity condition holds, $d^{\Gamma_{k}}$ is a strict interior point of $\left\{d^{\Gamma_{k}}|l_{\Gamma_{k}}-x_{k}^{\Gamma_{k}}\leq d^{\Gamma_{k}}\leq u_{\Gamma_{k}}-x_{k}^{\Gamma_{k}}\right\}$, and $\alpha_{k}^{*}$ is always positive (see [53] in detail).

Based on the above discussions, we state our algorithm as follows.

**Algorithm 1.** (Act-L-BFGS-Alt-Non)

**Step 0:** Given *x*_0_ ∈ Ψ, *ε*_0_ ∈ (0, 1), $0<\sigma <\frac{1}{2}$ and positive integer *m*, the “basic matrix” *θI*, set *k* = 0.

**Step 1:** Use (26) to determine Υ_*k*_ = Υ(*x*_*k*_), Λ_*k*_ = Λ(*x*_*k*_), and Γ_*k*_ = Γ(*x*_*k*_).

**Step 2:** Compute *d*_*k*_ by (28).

**Step 3:** If *d*_*k*_ = 0, stop.

**Step 4:** Choose 0 < *ε*_*k*+1_ < *ε*_*k*_ and *α*_*k*_ = 2^−*i*^, where *i* is the smallest integer of {0, 1, 2, …} such that the line search rule (23) holds.

**Step 5:** Let *x*_*k*+1_ = *x*_*k*_ + *α*_*k*_*d*_*k*_ and update *H*_*k*_ by (19).

**Step 6:** Set *k* ≔ *k* + 1 and go to Step 1.

## Global convergence

In order to prove global convergence of Algorithm 1, the following further assumption is needed.

**Assumption C.** There exist positive scalars *ς*_1_, *ς*_2_ such that any sequence of matrices ${\bar{H}}^{k},k=1,2,\ldots ,$ satisfy

$$\begin{array}{c} \varsigma_{1}{∥z∥}^{2}\leq z^{T}{\bar{H}}_{k}z\leq \varsigma_{2}{∥z∥}^{2},\forall \;\;z\in \;{ℜ}^{|\Gamma_{k}|}. \end{array}$$

The following lemma shows that *d*_*k*_ ≠ 0 is determined by (28) satisfies the sufficiently descent property.

**Lemma 1.** *Suppose that* *d*_*k*_ ≠ 0 *is determined by* (28) *and* *x*_*k*_ ∈ Ψ. *Then the inequality*

$$\begin{array}{c} g{\left(x_{k},\varepsilon_{k}\right)}^{T}d_{k}\leq -\omega {∥d_{k}∥}^{2} \end{array}$$ (32)

*holds with a constant* *ω* > 0.

**Proof.** We prove this result by three cases.

**Case 1.** *i* ∈ Υ_*k*_. By *x*_*k*_ ∈ Ψ and $d_{k}^{i}\neq 0,$ we get $d_{k}^{i}=l_{i}-x_{k}^{i}<0,$ implying *a*_*i*_(*x*_*k*_) > 0 and

$$\begin{array}{c} {\left(d_{k}^{i}\right)}^{T}g^{i}\left(x_{k},\varepsilon_{k}\right)\leq -\frac{{\left(d_{k}^{i}\right)}^{2}}{a_{i}\left(x_{k}\right)}\leq -\frac{{\left(d_{k}^{i}\right)}^{2}}{A_{i}}, \end{array}$$

where *A*_*i*_ is an upper bound on *a*_*i*_(*x*) in Ψ.

**Case ii.** *i* ∈ Λ_*k*_. As in Case i, it is easy to get $d_{k}^{i}=u_{i}-x_{k}^{i}>0,$ which means that *b*_*i*_(*x*_*k*_) > 0 and

$$\begin{array}{c} {\left(d_{k}^{i}\right)}^{T}g^{i}\left(x_{k},\varepsilon_{k}\right)\leq -\frac{{\left(d_{k}^{i}\right)}^{2}}{b_{i}\left(x_{k}\right)}\leq -\frac{{\left(d_{k}^{i}\right)}^{2}}{B_{i}}, \end{array}$$

where *B*_*i*_ is an lower bound on *b*_*i*_(*x*) in Ψ.

**Case iii.** *i* ∈ Γ_*k*_. By (29), (30), and with ${\bar{H}}_{k}$ symmetric positive definite, we obtain $d_{k}^{\Gamma_{k}}=-\alpha_{k}^{*}{\bar{H}}_{k}g_{k}^{\Gamma_{k}}\left(x_{k},\varepsilon_{k}\right).$ Then

$$\begin{array}{c} {\left(d_{k}^{\Gamma_{k}}\right)}^{T}{\left({\bar{H}}_{k}\right)}^{-1}d_{k}^{\Gamma_{k}}=-\alpha_{k}^{*}{\left(d_{k}^{\Gamma_{k}}\right)}^{T}g^{\Gamma_{k}}\left(x_{k},\varepsilon_{k}\right). \end{array}$$

By Assumption C, we have $\frac{1}{\varsigma_{2}}∥d_{k}^{\Gamma_{k}}{∥}^{2}\leq -\alpha_{k}^{*}{\left(d_{k}^{\Gamma_{k}}\right)}^{T}g^{\Gamma_{k}}\left(x_{k},\varepsilon_{k}\right)\leq \frac{1}{\varsigma_{1}}{∥d_{k}^{\Gamma_{k}}∥}^{2}$. So we have

$$\begin{array}{c} {\left(d_{k}^{\Gamma_{k}}\right)}^{T}g^{\Gamma_{k}}\left(x_{k},\varepsilon_{k}\right)\leq -\frac{1}{\rho_{2}\alpha_{k}^{*}}{∥d_{k}^{\Gamma_{k}}∥}^{2}. \end{array}$$

Letting $\omega =\min\{A_{i},B_{i},\frac{1}{\rho_{2}\alpha_{k}^{*}}\}$ completes the proof. □

The following lemma is similar to [52], so we state it without proof.

**Lemma 2.** *If the conditions in Lemma 1 hold*, *then* *x*^*k*^ *is a stationary point of* (3) *if and only if* *d*^*k*^ = 0. *Moreover*, $\bar{x}$ *is a stationary point of problem* (3) *when the subsequences* ${\left\{x_{k}\right\}}_{K}\to \bar{x}$ *and* {*d*_*k*_}_*K*_ → 0 *as* *k* → ∞.

Now we establish the global convergence theorem of Algorithm 1.

**Theorem 2.** *Let the sequence* {*x*_*k*_} *be generated by Algorithm 1 under Assumptions A*, *B*, *and C*. *Then the sequence* {*x*_*k*_} *at least has a limit point*, *and every limit point is a stationary point for problem* (3). □

**Proof.** If *d*_*k*_ = 0, by Lemma 2, the theorem obviously holds. Suppose that *d*^*k*^ ≠ 0. By Lemma 1, (23), and Assumption B, we obtain

$$\begin{array}{c} f^{MY}\left(x_{k}+\alpha_{k}d_{k},\varepsilon_{k+1}\right)\leq f^{MY}\left(x_{k},\varepsilon_{k}\right)+\sigma \alpha_{k}g{\left(x_{k},\varepsilon_{k}\right)}^{T}d_{k}\leq f^{MY}\left(x_{k},\varepsilon_{k}\right), \end{array}$$

which shows that the sequence {*f*^*MY*^ (*x*_*k*_, *ε*_*k*_)} is descending. So {*x*^*k*^} has at least a limit point. Suppose that $\bar{x}$ is a limit point of {*x*^*k*^}. It is sufficient to prove that $\bar{x}$ is a stationary point for problem (3). Lemma 2 means that we only show that the sequence {*d*_*k*_} → 0. Without loss of generality, we suppose that $\left\{d_{k}\right\}\to \bar{d}$ and $\left\{\varepsilon_{k}\right\}\to \bar{\varepsilon }$. By the property of limit, it is clear that $\bar{x}$ is feasible.

By the feasibility of $\bar{x}$, Theorem 1, and the positive functions *a*_*i*_(*x*) and *b*_*i*_(*x*) with any possible choice, we have

$$\begin{array}{c} {\left(\bar{x}-l\right)}_{\bar{\Upsilon }}=d_{\bar{\Upsilon }}=0,\;\;{\left(\bar{x}-u\right)}_{\bar{\Lambda }}=d_{\bar{\Lambda }}=0. \end{array}$$

It follows from (27) that

$$\begin{array}{c} g{\left(\bar{x},\bar{\varepsilon }\right)}_{\bar{\Gamma }}=0. \end{array}$$

By (30), we get $d_{\bar{\Gamma }}=0.$ Thus $d_{k}\to \bar{d}=0$. By Lemma 2, we deduce that $\bar{x}$ is a stationary point for problem (3).

**Remark.** If the condition $g(\bar{x},\bar{\varepsilon })=0$ holds, by (11) it is not difficult to deduce that $g(\bar{x})=0$ as $\bar{\varepsilon }\to 0$. By the convexity of *f*^*MY*^ (*x*), the point $\bar{x}$ is the optimal solution.

## Numerical results

In this section, we test the numerical behavior of Algorithm 1. All codes were written in MATLAB 7.6.0 and run on a PC Core 2 Duo CPU, E7500 @2.93GHz with 2GB memory and Windows XP operating system.

### Initialization

Our experiments are performed on a set of the nonlinear box-constrained nonsmooth problems from Karmitsa [54] which have given initial points. We choose *σ* = 0.1, *a*_*i*_(*x*) = *b*_*i*_(*x*) = 10^−5^ in (26), *θ* = 1 and the “basic matrix” to be the identity matrix *I* in the limited memory BFGS method, and *m* = 5. *ε*_*k*_ = 1/(*NF* + 1)^2^(*NF* is the function number). For subproblem (2), we use the PRP conjugate gradient algorithm, where the iteration number and the function number are added to the main program. Since the line search cannot always ensure the descent condition $d_{k}^{T}g_{k}<0,$ uphill search direction may occur in the numerical experiments. In this case, the line search rule may fail. In order to avoid it, the stepsize *α*_*k*_ is accepted if the search number is more than six in the line search. The following *Himmelblau* stopping rule isp used: If ∣ *f*^*MY*^ (*x*_*k*_, *ε*_*k*_) ∣ > 10^−4^, let $stop1=\frac{|f^{MY}\left(x_{k},\varepsilon_{k}\right)-f^{MY}\left(x_{k+1},\varepsilon_{k+1}\right)|}{|f^{MY}\left(x_{k},\varepsilon_{k}\right)|}$; Otherwise, let *stop*1 = ∣ *f*^*MY*^ (*x*_*k*_, *ε*_*k*_) − *f*^*MY*^ (*x*_*k*+1_, *ε*_*k*+1_) ∣. If *stop*1 < 10^−4^, the program stop. We also stop the program if the iteration number is more than 5000, and the corresponding method is considered to have failed.

### Results

In this section, the test results of our algorithm for some box-constrained nonsmooth problems are reported. The columns of Table 1 have the following meaning:

Dim: the dimension of the problem;    NI: the total number of iterations;

NF: the total number of function values;  cpu: the cpu time in second;

$f(\bar{x})$: denotes the function value at the point $\bar{x}$ when the program is stopped.

**Table 1:** Numerical results.

Problem	dim	NI/NF	$f(\bar{x})$	cpu
Generalization of MAXQ	1000	73/292	2.499289e-3	8.906250e-1
	2000	73/292	2.499289e-3	2.546875e+0
	3000	73/292	2.499289e-3	5.406250e+0
	5000	73/292	2.499289e-3	1.239063e+1
Generalization of MXHILB	1000	2/6	0	3.281250e-1
	2000	2/6	0	9.843750e-1
	3000	2/6	0	2.031250e+0
	5000	2/6	0	5.343750e+0
Chained LQ	1000	10/64	-8.060027e+2	5.468750e-1
	2000	10/64	-1.612812e+3	1.765625e+0
	3000	10/64	-2.419622e+3	3.890625e+0
	5000	10/64	-4.033241e+3	9.078125e+0
Chained CB3 I	1000	10/70	4.201502e+3	3.437500e-1
	2000	10/70	8.407210e+3	9.218750e-1
	3000	10/70	1.261292e+4	1.750000e+0
	5000	10/70	2.102433e+4	3.890625e+0
Chained CB3 II	1000	10/70	4.201502e+3	2.031250e-1
	2000	10/70	8.407210e+3	6.562500e-1
	3000	10/70	1.261292e+4	1.359375e+0
	5000	10/70	2.102433e+4	3.328125e+0
Number of active faces	1000	2/6	0	2.500000e-1
	2000	2/6	0	7.968750e-1
	3000	2/6	0	1.468750e+0
	5000	2/6	0	3.875000e+0
Generalization of Brown function 2	1000	59/236	9.913220e+1	8.906250e-1
	2000	59/236	1.983636e+2	2.484375e+0
	3000	59/236	2.975951e+2	4.765625e+0
	5000	59/236	4.960579e+2	1.032813e+1
Chained Mifflin 2	1000	13/52	-5.713089e+2	3.906250e-1
	2000	21/56	-1.143234e+3	1.140625e+0
	3000	20/52	-1.715185e+3	2.031250e+0
	5000	20/52	-2.859244e+3	5.062500e+0
Chained Crescent I	1000	59/236	1.448230e+2	7.812500e-1
	2000	59/236	2.897910e+2	2.171875e+0
	3000	59/236	4.347589e+2	4.500000e+0
	5000	66/236	7.246948e+2	9.578125e+0
Chained Crescent II	1000	59/236	1.448230e+2	7.031250e-1
	2000	59/236	2.897910e+2	2.078125e+0
	3000	59/236	4.347589e+2	4.546875e+0
	5000	66/236	7.246948e+2	9.234375e+0

The numerical results indicate that our algorithm is effective for these box constrained nonsmooth problems. The iteration number and function number do not change obviously with the increasing dimension. Problems Chained CB3 I and Chained CB3 II, and Chained Crescent I and Chained Crescent II, have many similar properties and have the same optimal values. From Table 1, we see that the final function value is close to the optimal value, especially for Problems Chained CB3 I and Chained CB3 II, and Chained Crescent I and Chained Crescent II, whose final function values are the same respectively, which shows that the presented method is stable. The cpu time is acceptable for this algorithm, although the iteration number is large for some problems. In the experiments, we find that different stopping rules influence the iteration number and the function number, but not the final function value.

**Fig 1:**
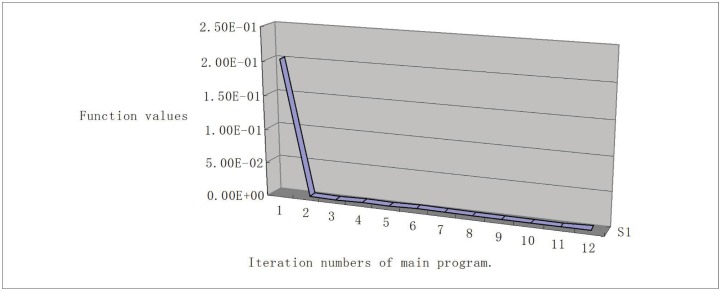
Generalization of MAXQ with 5,000 variables.

**Fig 2:**
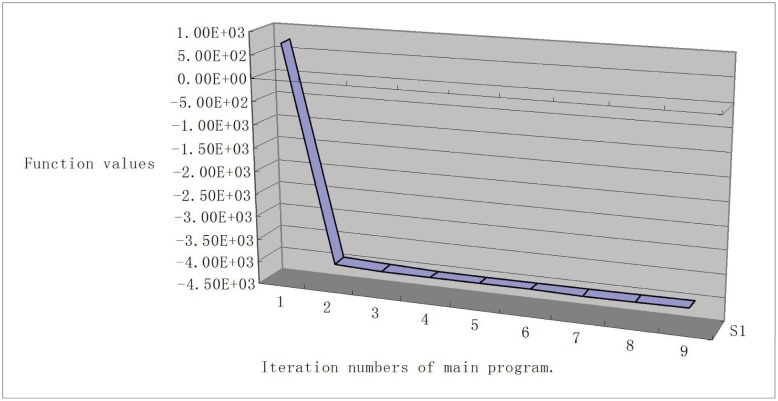
Chained LQ with 5,000 variables.

**Fig 3:**
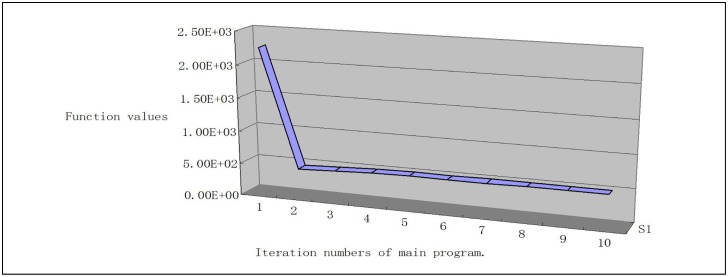
Generalization of Brown function with 5,000 variables.

**Fig 4:**
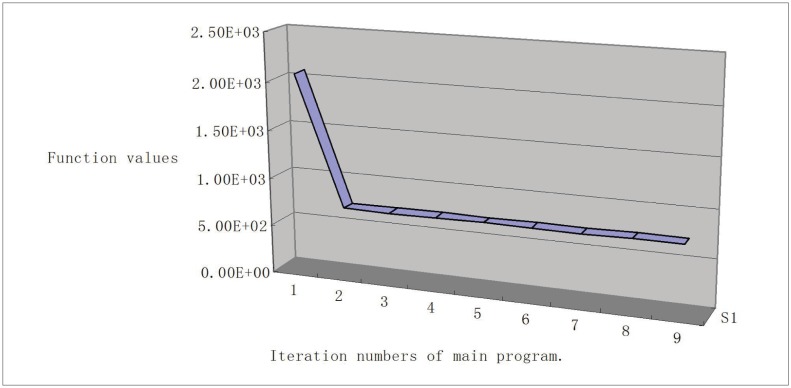
Chained Crescent I with 5,000 variables.

To show the sequence of function values, we give the line chart graph (Figs 1, 2, 3 and 4) for problems Generalization of MAXQ (Fig 1), Chained LQ (Fig 2), Generalization of Brown function 2 (Fig 3), and Chained Crescent I (Fig 4) withp 5,000 variables. We see that the functions are descending. The descent property of the first two steps is very obvious, and these two steps make the function value close to the optimal value. However, the descent is not obvious for other steps. In our opinion, the reason is that the stopping rules are not ideal. Overall, the numerical performance of the proposed algorithm is reasonable for these large-scale nonsmooth problems. We conclude that the method provides a valid approach for solving large-scale box-constrained nonsmooth problems.

## Conclusion

In this paper, a modified L-BFGS method was presented for solving box constrained nonsmooth optimization problems. This method uses both gradient information and function values in the L-BFGS update formula. The proposed algorithm possesses global convergence.

(i) It is well known that nonsmooth problems are difficult to solve even when the objective function is unconstrained, especially for large-scale nonsmooth problems. To overcome this drawback, the Moreau-Yosida regularization technique is proposed to make the objective function smooth. Moreover, the L-BFGS method is introduced to reduce the computation and make the active-set algorithm suitable for solving large-scale nonsmooth problems.

(ii) The bundle method is one of the most effective methods for nonsmooth problems. However, its efficiencies are applied to small- and medium-scale problems. In order to find more effective methods for large-scale nonsmooth problems, the bundle L-BFGS algorithms are presented by many scholars, where the dimension can be 1,000 variables. In this paper, the given algorithm can successfully solve 1,000-5,000 variables nonsmooth problems with bound constraints.

(iii) In experiments, we find the different stopping rules influence the iteration numbers and the function numbers but not the final functions. Moreover, from Figs 1–4, we see that the first two iteration steps are the most effective, which shows that the proposed algorithm is effective for large-scale nonsmooth box constrained problems. In our opinion, the reason lies in the stopping criteria. Better rules should be found.

(iv) Considering the above discussions, we think there are at least four issues that could lead to improvements. The first that should be considered is the choice of the parameters in the active-set identification technique. The parameters used are not the only choice. Another important point that should be further investigated is the adoption of the gradient projection technique. The third is adjustment of the constant *m* in the L-BFGS update formula. The last is the most important one, from the numerical experiments, namely whether are there other optimality conditions and convergence conditions in the nonsmooth problems? We will study these aspects in our future works.

Although the proposed method does not obtain significant development that we expected, we feel that its performance is noticeable.
